# Adrenal adenoma secreting 17-hydroxyprogesterone mimicking non-classical 21-hydroxylase deficiency

**DOI:** 10.3389/fendo.2024.1499836

**Published:** 2024-11-21

**Authors:** Beata Woźniak, Dorota Leszczyńska, Alicja Szatko, Karolina Nowak, Radosław Samsel, Anna Siejka, Lucyna Papierska, Wojciech Zgliczyński, Henrik Falhammar, Piotr Glinicki

**Affiliations:** ^1^ Department of Endocrinology, Centre of Postgraduate Medical Education, Warsaw, Poland; ^2^ EndoLab Laboratory, Centre of Postgraduate Medical Education, Warsaw, Poland; ^3^ Department of Surgery, Clinic of Surgical Oncology and Neuroendocrine Tumors, Maria Sklodowska-Curie National Research Institute of Oncology, Warsaw, Poland; ^4^ Clinical Biochemistry Department, The Children’s Memorial Health Institute, Warsaw, Poland; ^5^ Department of Endocrinology, Karolinska University Hospital, Stockholm, Sweden; ^6^ Department of Molecular Medicine and Surgery, Karolinska Institutet, Stockholm, Sweden

**Keywords:** congenital adrenal hyperplasia, CAH, adrenal incidentaloma, adrenal tumors, urine steroid profile, 17-OHP

## Abstract

In adrenal steroidogenesis, 17-hydroxyprogesterone (17-OHP) is a substrate for 21-hydroxylase, one of the crucial enzymes of the cortisol and aldosterone biosynthesis pathway. Thus, measurement serum 17-OHP concentration is used when the diagnosis of congenital adrenal hyperplasia (CAH) due to 21-hydroxylase deficiency is suspected. In the classic 21-hydroxylase deficiency, randomly timed measurements of 17-OHP are generally significantly elevated using different immunoassays. In the non-classic form of CAH (NC-CAH), the activity of 21-hydroxylase is less decreased, therefore the measurements of 17-OHP after ACTH stimulation test are usually required for diagnosis. Nonetheless, elevated 17-OHP concentration may also origin from adrenal tumors or ovarian neoplasms as a result of defects in steroidogenesis with an accumulation of steroids precursors. The presented cases and the literature review draw attention to the occurrence of rare causes of benign adrenal adenomas with steroidogenesis defects which may lead to a misdiagnosis of CAH.

## 1 Introduction

Congenital adrenal hyperplasia (CAH) belongs to a group of genetic diseases, inherited in an autosomal recessive manner. The most common cause of CAH, found in 90%-99% of cases, is 21-hydroxylase (21-OH, *CYP21A2*) deficiency (21-OHD) (1). The CYP21A2 enzyme (EC 1.14.99.10) catalyzes the conversion of 17α-hydroxyprogesterone (17-OHP) to 11-deoxycortisol and progesterone to 11-deoxycorticosterone (DOC), which provide a substrate for other enzymes that convert these steroids leading to production of aldosterone and cortisol. 21-OHD, depending on the type of gene variant, can have various phenotypes, from severe to mild or asymptomatic (2). The gene encoding 21-OH is located on chromosome 6 (6p21.3) in the human leukocyte antigen (HLA) gene region (3). 21-OHD may result from deletions, conversions and point variants in the *CYP21A2* gene. Micro conversions occur in approximately 85% (4). Depending on the *CYP21A2* gene variant, which translates into the degree of 21-OHD, there are three types of the disease: classic with salt-wasting, classic without salt-wasting (simple virilization) and non-classic (NC) type. The highest 17-OHP concentrations in serum are observed in the classic CAH. The concentration of 17-OHP in the NC form only slightly exceeds the upper references values. Serum cortisol concentration is reduced, although in cases with NC-CAH it may be within the reference range. Serum androgen concentrations (e.g. androstenedione, testosterone) most often exceed references values in all phenotypes, although the more severe phenotype the higher concentrations (5).

Basal serum 17-OHP concentrations >10 ng/ml (>30 nmol/L) indicate CAH, although lower basal concentrations are usually present in NC-CAH (6). In these cases, 17-OHP after ACTH (adrenocorticotropic hormone) stimulation is needed. The clinical features observed in patients with NC-CAH are subtle and include mild to moderate hirsutism, acne, precocious puberty, menstrual disorders and impaired fertility (5). NC-CAH may resemble other diseases, including PCOS (polycystic ovary syndrome) which is the most common cause of hyperandrogenism in women. It should be noted that PCOS is a diagnosis of exclusion. NC-CAH is not uncommon, and the incidence may be as high as 1 in 200 people (7).

The co-occurrence of adrenal tumors and CAH is known. Elevated ACTH plasma concentrations, most often due to untreated or insufficiently treated CAH, are considered a factor driving the growth of adrenocortical cells, leading to the formation of adrenal tumors (8). It is estimated that 0.8%–5.9% of adrenal incidentalomas are caused by undiagnosed CAH (9). However, very few physicians exclude undiagnosed CAH in the work-up of an adrenal tumor (10).

The serum concentration of 17-OHP may also be increased in malignant and benign adrenocortical tumors, or in bilateral macronodular adrenal hyperplasia, therefore the results should be interpreted approached with caution (11). If high 17-OHP concentrations are found, especially at concentrations between 10 ng/ml (30 nmol/L) and 20 ng/ml (60 nmol/L), genetic testing for *CYP21A2* mutations should be performed (9). In cases with adrenal tumors and increased 17-OHP concentrations and other steroid precursors, low or decreased ACTH concentrations suggest against a CAH diagnosis.

There are only a few reports in the literature of 17-OHP production by benign adrenal tumors, which may be the result of intratumoral 21-OHD. Thus, we present the diagnostic challenges in two women with elevated 17-OHP concentrations and adrenal tumors, initially diagnosed with NC-CAH, whose 17-OHP concentrations normalized after adrenalectomy.

## 2 Case reports

### Patient 1

**Figure 1:**
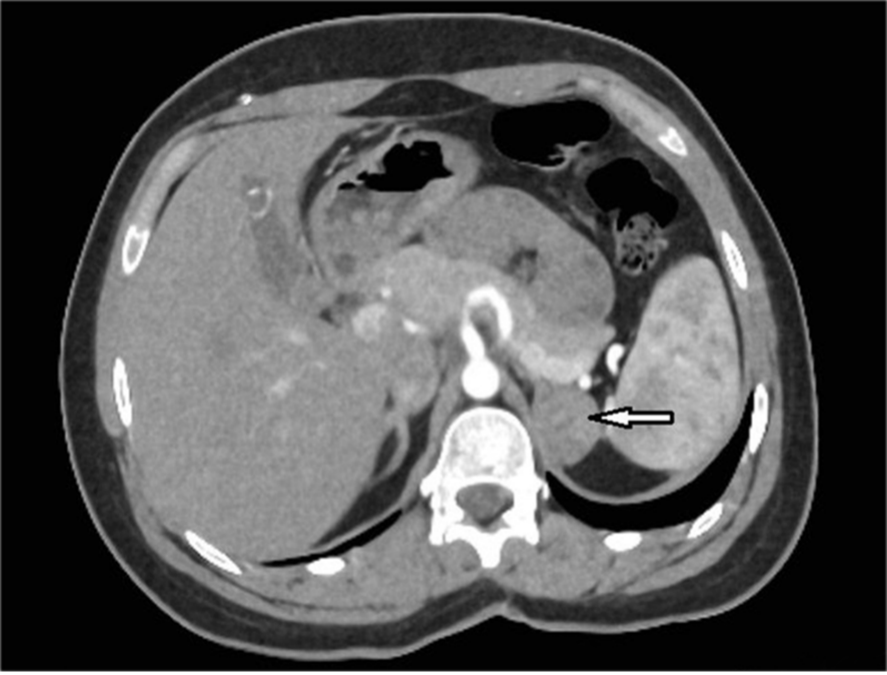
An axial abdominal computed tomography (CT) scan with intravenous contrast administration of case 1 revealed an indeterminate mass communicating with the left adrenal gland measuring approximately 28 x 21 mm (arrow).

A 22-year-old woman was referred to the Department of Endocrinology due to a focal lesion in the left adrenal gland. She complained of oligomenorrhea and acne. Her previous history included flaccid paresis of the lower limbs as a result of myelomeningocele in the lumbar-sacral spine after the insertion of a ventriculoperitoneal shunt due to hydrocephalus in childhood. She was not married and had no children. The patient’s first symptoms of puberty appeared before she was 8 years old. Since then, she had oligomenorrhea. For this reason, from about the age of 16, she periodically took progesterone to regulate her cycles. Her history also revealed that she had been hospitalized twice in the gynecological ward due to menstrual disorders, where a basal 17-OHP concentration of 5.8 ng/ml (17.4 nmol/L) had been found. On ACTH stimulation test, 30 and 60 minute 17-OHP concentrations were 31.4 ng/ml (95.0 nmol/L) and 31.2 ng/ml (94.4 nmol/L), respectively. Other laboratory tests performed on days 3-5 of the menstrual cycle showed normal androgen concentrations (**Table 1**). Other laboratory tests are listed in **Table 2**. Based on these results, she was diagnosed with NC-CAH. The computed tomography (CT) showed an indeterminate mass communicating with the left adrenal gland measuring 28 x 21 mm (**Figure 1**). The density of the lesion in the native phase was 37 HU (Hounsfield unit) with no visible fat values. The gynecological ultrasound did not reveal any adnexal tumors or polycystic features, the uterine body was homogeneous and of normal size.

**Table 1:** Hormonal Assessment.

Hormones in serum or plasma	Patient 1 before operation	Patient 1 after operation	Patient 2 before operation	Patient 2 after operation	Reference range
Cortisol (8.00 a.m.), μg/dl	12.6	20.5	25.7	12.8	5-25
24-hr UFC, μg/24hr	116.8	50.6	N/A	N/A	20.0–130.9
ACTH, pg/ml	16.5	54.6	25.9	15.9	5-46
Aldosterone, ng/dl	7.80	20.80	17.1	7.44	2.52-39.2
Renin (DRC), μIU/ml	13.70	44.13	269.3	>500	4.4-46.1
DHEA-S, mg/dl	123	87	36	37	80.2–339
Androstenedione, ng/ml	1.97	1.39	0.48	0.48	0.4-3.4
17-OHP, ng/ml	14.3	0.97	25.5	0.47	< 2.0
Testosterone, ng/ml	0.220	0.140	0.29	<0.07	0.1-0.7
SHBG, nmol/L	62.4	60.9	54.5	35.2	48.2-137.2
TSH, μIU/ml	4.815	3.531	1.06	2.358	0.35-4.0
LH, IU/L	2.3	N/A	39.0	38.5	1.1-9.2
FSH, IU/L	7.0	N/A	124.0	150.0	2.8-10.2
Estradiol, pg/ml	35.2	90.0	<10.0	10.1	0-112.0
Prolactine, ng/ml	7.6	N/A	11.8	8.6	1.9-25.0
Chromogranin A (CgA), ng/ml	69.6	N/A	N/A	N/A	20-98
Plasma free metanephrine, pg/ml	43.1	N/A	70.89 *	N/A	< 62.0
Plasma free normetanephrine, pg/ml	51.4	N/A	37.8 **	N/A	< 124.0
Plasma free 3-methoxytyramine, pg/ml	< 5	N/A	<10	N/A	< 10.0

UFC, urinary free cortisol; N/A, not available; DRC, direct renin concentration; SHBG, sex hormone binding protein; TSH, thyroid-stimulating hormone; DHEA-S, dehydroepiandrosterone sulfate; 17-OHP, 17-hydroxyprogesterone; LH, luteinizing hormone; FSH, follicle-stimulating hormone; ACTH, adrenocorticotropic hormone; * plasma free metanephrine, reference value < 88 pg/ml; ** plasma free normetanephrine, reference value < 168 pg/ml.

**Table 2:** Urine Steroid Profile (GC/MS) before and after adrenalectomy.

Urine steroid profile, μg/24hr	Patient 1 before operation	Patient 1 after operation	Patient 2 before operation	Patient 2 after operation	Reference range
AN (androsteron)	574.1	803.1	278.3	422.3	460-3660
ET (etiocholanolone)	525.8	892.9	370.2	466.4	490-3680
11-OAN/ET (11-ketoandrosterone/etiocholanolone)	131.4	181.9	229.8	159.5	123-990
11-OHAN (11-hydroxy-androsterone)	**1715.9 ↑**	668.1	726.1	593.6	200-1190
11-OHET (11-hydroxy-etiocholanolone)	71.1	143.7	191.7	241.9	24-845
DHA (dehydroepiandrosterone)	390.2	531.2	10	32.6	34-1500
5-AND (5-androstendiol)	**349.8 ↑**	227.3	21.7	96.5	29-230
16a-OHDHA (16alpha-hydroxy-DHA)	**1469.4 ↑**	**1573.8 ↑**	48.3	52.7	63-1380
An-3-ol (5-androstentriol)	145.6	182.5	38.1	74.1	90-720
5-PT (5-pregnentriol)	263.3	296.7	34	74.1	60-360
17-OHPN (5beta) (17-hydroxy-pregnanolone)	**2339.5 ↑**	82.8	**2617.4 ↑**	67.2	63-280
17-OHPN (5alpha) (17-hydroxy-pregnanolon)	130.7	5.5	61.1	2.3	N/A
PT (pregnantriol)	**3064.9 ↑**	419.1	**2701.8 ↑**	380.4	179-992
PTN (pregnantriolone)	**1888.9 ↑**	42.3	**592.2 ↑**	16.3	3,5-50
PD (pregnandiol)	487.3	200.4	**1507.5 ↑**	166	<900

N/A, not available; ↑, above upper reference range. ↑, above upper reference range

A magnetic resonance imaging (MRI) similarly showed a focal lesion in the left adrenal gland with no attenuation of the signal in antiphase. Taking into account the indeterminate adrenal tumor and the young age of the patient, adrenalectomy was performed.

Histopathological examination of the adrenal tumor revealed an epithelial cell tumor without obvious atypia, vascular invasion nor necrosis and with a low mitotic index (1fp/10HPF). The microscopic image and immunohistochemistry profile [inhibin (+), Melan A (+), synaptophysin (+), chromogranin A (-), S100 (-), Ki-67 index (+) 1%] confirm the diagnosis of adrenocortical adenoma. During follow-up examinations 4 months and 1 year after the surgery, the serum 17-OHP concentrations normalized, and the urine steroid profiling, apart from slightly increased excretion of one of the dehydroepiandrosterone metabolites (16-alpha-hydroxy-DHA), showed normal daily excretion of the adrenal androstenedione metabolite, 17-OHP metabolites and 21-deoxycortisol were found (**Table 2**).

### Patient 2

A 57-year-old woman, diagnosed with Hodgkin disease 30 years ago, presented to the Department of Endocrinology due to a 35 x 33 mm right sided adrenal incidentaloma with 25 HU on unenhanced CT performed due to abdominal pain. Laboratory tests performed at another hospital showed high 17-OHP concentrations and based on results from ACTH stimulation test she had been diagnosed with NC-CAH. Upon admission to the Department of Endocrinology she did not present any symptoms of hyperandrogenism. Except for increased concentration of basal 17-OHP (>11 ng/ml/>33 nmol/L, after dilution (1:10) 25.5 ng/ml/77.2 nmol/L) while other biochemical tests including dexamethasone suppression test (DST) were normal (**Table 1**). Steroid urine profile revealed elevated concentration of 17-OH-progesteron metabolites which was consistent with NC-CAH diagnosis (**Table 2**). MRI with chemical shift showed a nonhomogeneous tumor without loss of signal intensity on out-phase imaging. Due to indeterminate character of the adrenal tumor with suspected isolated secretion 17-OHP the patient underwent adrenalectomy. Histopathological examination showed only low-grade nuclear atypia with other typical characteristics of adrenocortical adenoma (no necrosis, low IM 1/HPF, Ki-67 index: 1%-2%, no venous, sinusoid or capsular invasion). The patient missed her first follow-up visit due to breast cancer surgery (ductal carcinoma *in situ*). The patient came for an appointment 1 year later and then both serum 17-OHP concentrations (0.7 ng/ml/2.2 nmol/L) as well as steroid urine profiling had normalized.

## 3 Discussion

CAH due to 21-OHD is characterized by an excess of progesterone, 17-OHP, DHEAS (Dehydroepiandrosterone sulfate), androstenedione and testosterone accompanied by varying degrees of mineralocorticoid and cortisol deficiencies. Despite high 17-OHP concentrations, our patients had normal testosterone, androstenedione, DHEAS, aldosterone and cortisol concentrations. Based on the results of the urinary steroid profile, increased excretion of adrenal androstenedione metabolites, 17-OHP and 21-deoxycortisol metabolites with an increased ratio to cortisol metabolites was demonstrated. Therefore, both cases were diagnosed with NC-CAH, however, after adrenalectomy the increased 17-OHP concentrations normalized suggesting increased 17-OHP production by the adrenal tumor.

Due to the widespread use of imaging modalities, the detection rate of adrenal incidentalomas is approximately 4.5% (12). After finding each such lesion exceeding 1 cm in diameter, in-depth diagnostics should be performed. This is to distinguish benign adenomas, which most often do not have hormonal activity, from malignant tumors or pheochromocytomas (11). An increased 17-OHP concentration may suggest a case of CAH or an intrinsic effect of the adrenal tumor. The urinary steroid profile is useful in differentiating between them (13, 14) and could therefore be considered in every patient with an indeterminate adrenal mass to help guide further diagnostic and therapeutic measures. During the analysis, it is possible to determine the metabolites of all steroid precursors in the biosynthetic pathway of cortisol, aldosterone and sex hormones. Moreover, it has been recommended that all patients diagnosed with NC-CAH should be genetically confirmed with a *CYP21A2* analysis to avoid misdiagnosis (5).

Another situation mimicking NC-CAH, which has been repeatedly described, is hyperandrogenemia with elevated 17-OHP concentrations in the setting of ovarian steroid cell tumors (SCT) (15–18). Undiagnosed CAH, poor compliance with medical recommendations, Nelson syndrome or other associations with excessive ACTH secretion may lead to the formation of SCTs as ovarian and others adrenal rest tumors.

The cases we have described also indicate the need for diagnostics for hormonally active adrenal tumors in which the enzymatic activity of cancer cells has been changed as a feature of dedifferentiation. The association of excessive 17-OHP response to ACTH in adrenal tumors has long been studied (9). In most published studies, researchers concluded that in the case of adrenal tumors there may be a significant increase in 17-OHP in the ACTH stimulation test compared to the control group, although they did not record values exceeding 5 ng/ml (15 nmol/L) (19–21). Only one study included patients whose initial 17-OHP concentrations exceeded references values (2 ng/ml/6 nmol/L) or whose post-stimulation values exceeded 10 ng/mL (30 nmol/L) (22). In the majority of study subjects who were treated surgically, their 17-OHP concentrations normalized after the procedure, which confirms the above assumptions. Moreover, in some reports, there was no correlation between the size of the adrenal gland mass and the amount of increase in 17-OHP concentration (20, 23). However, most other studies showed a positive correlation between ACTH-stimulated serum17-OHP concentrations and the size of non-functioning adrenal tumors (9, 24–26).

If we compare the frequency of intratumoral 21-OHD in cases of unilateral and bilateral tumors, an excessive response of 17-OHP was more often observed in the case of bilateral lesions (66.6%) than in unilateral tumors (50%) (27, 28). These data indicate that intratumoral 21-OHD is very common in adrenal tumors and that the likelihood of this enzymatic defect will be greater in bilateral than unilateral lesions, although in all presented cases in these two studies the defect in steroidogenesis was not significant enough to misdiagnose CAH but it has probably been done in other published cases (9).

In histologically different types of tumors, steroidogenesis may be impaired. These are both benign and malignant lesions, including adenomas, myelolipomas, lymphomas, hamartomas, carcinomas and even pheochromocytomas (20, 23, 24). This means that we are unable to assess the nature of the lesion based on secretory activity. Occasionally, there may be a transient overproduction of 17-OHP by the tumor, manifested by a transient partial intratumoral 21-OHD with increased 17-OHP concentration in the active phase of adrenal tumor (29, 30).

Not only can we encounter intratumoral defects in 21-OH, but also P450c11 (11β-hydroxylase) deficiency in the adenoma can cause steroid abnormalities mimicking CAH (31, 32). The increased amplitude of the response to ACTH-stimulation in terms of other testable steroid precursors (progesterone, 17-OHP, 11-deoxycorticosterone, corticosterone and 11-deoxycortisol) may confirm reduced activity of steroidogenic enzymes such as CYP11B1, CYP11B2 (28, 33, 34).

All these observations confirm the possibility of the presence of potential intratumoral deficits of various steroidogenic enzymes.

## 4 Conclusions

There are various forms of steroidogenesis disorders occurring in both benign and malignant tumors. Therefore, steroid tests results should be interpreted with caution and all possible causes should be taken into account when making the final diagnosis. In the case of hyperandrogenemia, extensive hormonal diagnostics (different immunoassays) should always be performed, including imaging of the adrenal glands and ovaries using various methods (eg. CT, MRI) and should be supported by steroid profile in the urine or plasma (GC/MS or LC-MS/MS, Liquid Chromatography–Mass Spectrometry) or molecular testing. A diagnosis of CAH should ideally be genetically confirmed, especially in the presence of adrenal tumors.
